# Temperature Tolerance and Thermal Environment of European Seed Bugs

**DOI:** 10.3390/insects11030197

**Published:** 2020-03-20

**Authors:** Helmut Käfer, Helmut Kovac, Nikolay Simov, Andrea Battisti, Bettina Erregger, Arne K. D. Schmidt, Anton Stabentheiner

**Affiliations:** 1 Institute of Biology, University of Graz, 8010 Graz, Austria; 2 National Museum of Natural History, 1000 Sofia, Bulgaria; myrmedobia@gmail.com; 3 School of Agricultural Sciences and Veterinary Medicine, University of Padova, 35122 Padova, Italy; andrea.battisti@unipd.it; 4 Institute of Animal Nutrition, Livestock Products, and Nutrition Physiology, University of Natural Resources and Life Sciences, 1180 Vienna, Austria; bettina.erregger@gmail.com; 5 AGES, The Austrian Agency for Health and Food Safety, 1220 Vienna, Austria; arne.kd.schmidt@gmail.com

**Keywords:** true bugs, thermal limits, distribution, bioclimatic parameters, climate

## Abstract

Heteroptera, or true bugs populate many climate zones, coping with different environmental conditions. The aim of this study was the evaluation of their thermal limits and derived traits, as well as climatological parameters which might influence their distribution. We assessed the thermal limits (critical thermal maxima, CT_max_, and minima, CT_min_) of eight seed bug species (Lygaeidae, Pyrrhocoridae) distributed over four Köppen–Geiger climate classification types (KCC), approximately 6° of latitude, and four European countries (Austria, Italy, Croatia, Bulgaria). In test tubes, a temperature ramp was driven down to −5 °C for CT_min_ and up to 50 °C for CT_max_ (0.25 °C/min) until the bugs’ voluntary, coordinated movement stopped. In contrast to CT_min_, CT_max_ depended significantly on KCC, species, and body mass. CT_max_ showed high correlation with bioclimatic parameters such as annual mean temperature and mean maximum temperature of warmest month (BIO5), as well as three parameters representing temperature variability. CT_min_ correlated with mean annual temperature, mean minimum temperature of coldest month (BIO6), and two parameters representing variability. Although the derived trait cold tolerance (TC = BIO6 − CT_min_) depended on several bioclimatic variables, heat tolerance (TH = CT_max_ − BIO5) showed no correlation. Seed bugs seem to have potential for further range shifts in the face of global warming.

## 1. Introduction

Heteroptera, or true bugs, are a widely distributed, highly diverse insect taxon with approximately 45,300 [1] species worldwide. They populate every climate zone from arctic to desert [2,3,4], showing great ability to cope with a variety of environmental factors. This leads to a substantial potential for invasiveness, and indeed many bug species are known to be highly invasive. For example, *Halyomorpha halys*, an Asian stink bug invading Europe and North America, made it to the top four “most wanted” species list in the USA, inflicting economic damage of USD 37 million to tree fruit production in 2010 [5]. However, although some species thrive over wide latitudinal ranges, other closely related species are more restricted in their distribution range (e.g., *Orsillus depressus*: Southern and Central Europe, northwards until Scandinavia; *O. maculatus*: Southern Europe, around the Mediterranean and Black Sea) [6]. Species such as *Oxycarenus lavaterae* are known to have spread from their Mediterranean origins rather recently and are on their way north- and eastwards [7,8,9,10,11]. They seem to benefit from climate change, with its higher annual mean temperatures, as well as human behavior, which facilitates dispersion (often along traffic routes) and settlement (e.g., through extensive plantings of *Tilia* trees, a host plant of *Oxycarenus* in urban environments).

Temperature is a main parameter for thriving and spread of ectothermic insects [12,13,14]. Within the insects’ favorable temperature zone, it determines development and reproduction. Temperatures outside of this favorable zone hinder and become lethal at a certain point [15]. In temperate climate regions, the change from favorable to unfavorable is mainly a seasonal occurrence, with low temperatures during the winter and possible high temperature extremes during summer. Adaptations such as hibernation or winter diapause, and aestivation lets the organisms cope with these adverse environmental conditions.

The climate variability hypothesis states that organisms exposed to higher variations in their thermal environment show more tolerance to extreme temperatures [16]. In this regard, the organisms’ critical thermal maxima show less variation than their critical thermal minima [17,18,19]. Geographical latitude, seasonality in temperature, and also elevation above sea level are thought to correlate with the animals’ temperature tolerance breadth and be a valid measurement for climate variability (see for instance [18,20,21,22,23]).

Several attempts have been made to assess and model the distribution of ectotherms and endotherms in connection with thermal tolerance and macro-ecological scales such as climate parameters and latitude [18,20,24,25,26,27,28]. One problem correlating thermal traits of animals with climatic measurements are the differences in scale (climate data point = animal size × 10,000 [29]). Contradictory hypotheses regarding the species’ susceptibility to climate warming (e.g., [30] vs. [31]) show that large-scale climate-based studies might be insufficient to describe and predict biological effects. The assessment of microclimatic habitats as buffers of environmental conditions seems to be more suitable. However, even microclimate measurements do not always show the temperatures the animals are exposed to [32,33], because in reaction to uncomfortable conditions the animals show various physiological (e.g., evaporation [34,35,36]) and/or behavioral reactions (e.g., by burrowing or simply moving away [37,38]). Such micro-scale measurements, however, are often not feasible, or data are not accessible. For medium to large scale assessment of physiological and behavioral factors driving insect distribution and dispersion, therefore, standard meteorological data and their bioclimatic classifications are often the only accessible data source to study physiological and behavioral responses in order to gain a deeper understanding of the underlying mechanisms of animal survival under extreme environmental conditions [39]. Critical thermal limits are commonly used physiological parameters to assess factors driving insect dispersion and distribution. One way to describe upper and lower thermal limits of organisms is the definition of temperature when voluntary, coordinated movement stops [40,41,42], rendering the animal finally unable to react to an adverse environment via locomotion (i.e., moving away to another, more habitable part of its surroundings). 

The aim of this study was the evaluation of thermal tolerance traits (critical thermal maximum and minimum) of some common bugs from the temperate central Europe and the neighboring Mediterranean climate zones. The data were analyzed in relation to climatological and geographical parameters, as well as phylogenetic traits. With this knowledge, we wanted to test whether the climate variability hypothesis is applicable for this insect group, and whether their thermal traits are the basics for their high potential of invasiveness.

## 2. Materials and Methods

### 2.1. Animals

**Figure 1:**
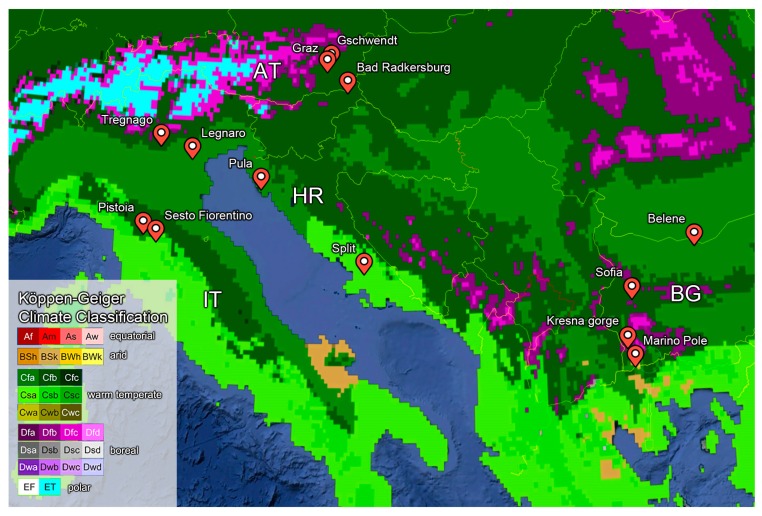
Sample locations and climate regions according to the Köppen–Geiger climate classification (KCC); map modified from Google Earth Pro, KCC overlay from http://koeppen-geiger.vu-wien.ac.at/.

Eight bug species common in Europe (Table 1) were sampled in four Köppen–Geiger climate (KCC) regions in Austria, Italy, Croatia, and Bulgaria during the cold season, from autumn (end of September) to winter (mid of January) in the years 2013, 2014, 2016, and 2018 (Figure 1, Table 2, Table S1). These bug species are distributed over wide areas of Europe, some restricted to the southern and central parts, and some spread to regions north of the polar circle [43]. All physiological measurements were performed on adult individuals at the laboratory in Austria. The animals were transported to the laboratory by ourselves or were delivered via express mail within 3 to 5 days. The bugs were sampled randomly at the respective sample sites. Sex of the individuals was not determined. All individuals were similar in their external characteristics (habitus, size, body mass). Therefore, we assume that we mapped a natural gender relationship of the respective populations. Individuals that were noticeably behaviorally impaired (possibly by transport) were excluded from the evaluation. This as well as different numbers of sampled individuals resulted in an inconsistent number (*n*) per species/sample site/experiment. All experiments were conducted immediately after the individuals arrived in our laboratory. All bug sampling, transportation, and experiments took place in concurrence with national and EU regulations.

### 2.2. Critical Thermal Minimum (CT_min_)

We placed the true bugs (see Table 2 for *n*) in individual measurement chambers (acrylic glass test tubes, about 3 to 10 ml, depending on size, which were mounted in a self-constructed shaking device. The device was submerged into a temperature-controlled water bath (JULABO F33, JULABO Labortechnik GmbH, Seelbach, Germany). Up to nine individuals of the same species were tested per trial run. An additional empty tube contained a thermocouple connected to a data logger (ALMEMO 2690, Ahlborn GmbH, Holzkirchen, Germany), which recorded the exact temperature the animals were exposed to in one second intervals. After 10 min of habituation at 15 °C, we drove a temperature ramp at a slope of −0.25 °C/min down to −5 °C. After 5 min at this temperature, we warmed the chambers at a rate of 2 °C/min until 15 °C was reached again. The measurement chambers were shaken forcefully by the shaking device once a minute for 1 s for the entire experiment (approximately 100 min). The experiments were recorded via video camera (Sony HDR-CX730E, Sony Europe Limited, Vienna, Austria) for later evaluation of the animals’ behavior. To determine the lower threshold of activity, the last appearance of voluntary movement of antennae or legs at a shaking event was determined. The temperature at the next shaking event was specified as the CT_min_ (according to [44]). All evaluated individuals survived the experiment and regained full mobility.

### 2.3. Critical Thermal Maximum (CT_max_)

We used a similar experimental setup as in Section 2.2, without the shaking apparatus activated. Stimulation was not necessary, as the insects were always active at higher temperatures. Up to 10 measurement chambers were used for the bugs; the thermocouple for measuring the temperature was placed in an additional similar chamber, near the animals. Following a standardized method (e.g., [45,46,47]), the experiments started at a temperature of 25 °C. After 10 min of habituation, we drove a temperature ramp up to 50 °C at a rate of 0.25 °C/min. Again, the animals’ behavior was recorded via video camera for later evaluation. The cease of coordinated movement marked the so-called knockdown point the experimental ambient temperature measured at this time was described as CT_max_ ([40,41,42]). As we did not abort the experiment after the first individual reached CT_max_ but finished the temperature ramp, no individuals survived the CT_max_ experiments.

### 2.4. Thermal Tolerance Breadth (TTB), Heat Tolerance (TH), Cold Tolerance (TC)

Thermal tolerance breadth (TTB) is the temperature range in which the bugs were able to exhibit voluntary coordinated movement. It was calculated as TTB = CT_max_ − CT_min_. The heat tolerance (TH) and cold tolerance (TC) were calculated from the upper and lower thermal limits and bioclimatic variables: TH = CT_max_ − BIO5 (maximum temperature of the warmest month), and TC = BIO6 − CT_min_ (BIO6 = minimum temperature of the coldest month), respectively. Positive values for TH and TC show the bugs’ ability to endure the occurring ambient temperatures. Negative values indicate that they will have to avoid heat or cold damage by other means. TTB, TH, and TC were calculated from mean CT_min_ and CT_max_ values per species and sample location.

### 2.5. Climate Data, Bioclimatic Variables, and Data Analysis

The climate regions of our study animals were detailed from the Köppen–Geiger climate classification system (KCC, Figure 1) updated by Kottek et al. [48]. The animal data were correlated with eight bioclimatic variables (BIO1 to BIO7; BIO12) extracted from the WorldClim dataset ([49], http://worldclim.org/, January 2019) using ArcGIS ArcMap 10.5 (Environmental Systems Research Institute, Redlands, CA, USA). The variables are the climatological normal from 1970–2000. The climatological normal is a 30 year average of a weather variable and is used as an average or baseline to evaluate climatic effects. Correlations of physiological data and climate variables were tested via ANOVA and general linear model (GLM) modules in STATGRAPHICS Centurion 18 (Statgraphics Technologies Inc., The Plains, Virginia, USA).

### 2.6. Testing for a Phylogenetic Signal

A detailed phylogenetic cladogram was not available for our sampled bug species. To model phylogenetic relations between the species, we generated a branching tree using an established taxonomy ([6,43], Table 1), which is preferable to ignoring taxonomic relationship entirely [50]. For the analyses regarding the phylogenetic signal, we built a phylogenetic tree based on recent studies of Heteroptera: Pentatomomorpha [51] using Mesquite software 3.6 [52]. The taxonomic positions of relevant subfamilies in this study were taken as template for the eight species (19 populations) assessed in our study, where polytomies were randomly resolved into a series of dichotomies. Due to missing exact relationships between species, we set branch length to 1 ([53]; see Figure S1). To incorporate phylogenetic signals (i.e., related species that have similar traits) for all temperature parameters, species, and body mass, we calculated Pagel’s λ [54] using R 3.5.1 [55]. The Pagel’s λ ranges between 0 (no phylogenetic signal) and 1 (strong phylogenetic signal) and was determined using the pgls function in the caper package [56]. 

## 3. Results

The investigated seed bugs showed high interspecies variability in their thermal traits. Mean CT_max_ ranged from 40.8 ± 0.6 °C in *Scolopostethus pictus* (Bad Radkersburg, AT, Cfb climate type) to 48.9 ± 0.5 °C in *Melanocoryphus albomaculatus* (Pistoia, IT; Cfa climate type, see Figure 1). CT_min_ ranged from −4.3 ± 0.2 °C in *S. pictus* (Bad Radkersburg, AT, Cfb) to −0.9 ± 0.6 °C in *Pyrrhocoris apterus* (Split, HR, Csa; mean values of individuals in a distribution area). TTB ranged from 45.1 °C (*Orsillus maculatus*, HR) to 52.0 °C (*M. albomaculatus*, IT). In addition, intraspecies variations were high in some cases. For details, see Table 2 and Figure S3.

### 3.1. Critical Thermal Limits (CT_min_, CT_max_)

Critical thermal maxima differed significantly among bug species (ANOVA, *F*-quotient = 387.82, *p* < 0.0001), with the exception of *Arocatus longiceps* and *Orsillus maculatus*. Critical thermal minima (ANOVA, *F*-quotient = 1.36, *p* > 0.05) did not differ significantly among bug species. 

**Figure 2:**
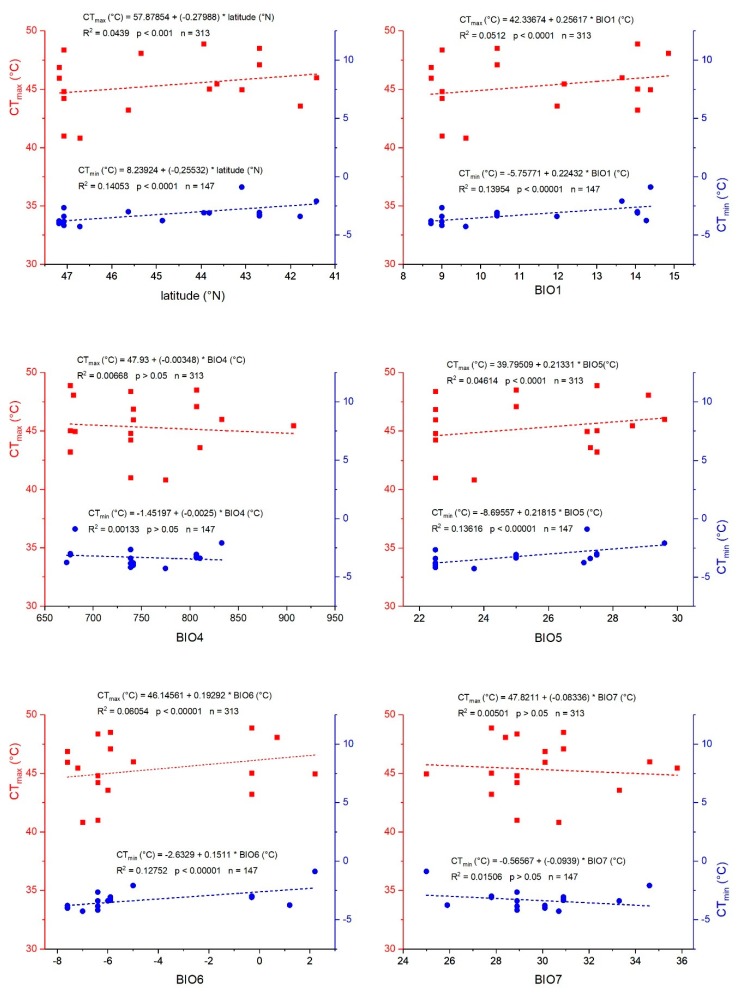
Correlation of physiological parameters CT_min_ (blue) and CT_max_ (red) with latitude and bioclimatic variables (BIO1, BIO4–BIO7; from http://worldclim.org/version2 [49]; variables are the average from 1970 to 2000). Dots represent means of individual species at certain sample sites. Correlations calculated with number of individuals. See also Table 2 and Table S2.

A multifactorial ANOVA with mass as covariate revealed a correlation of CT_min_ with KCC, but not with species or body mass (Table 3). The hotter the climate, the lower the tolerance against low temperatures was observed. General linear model (GLM) statistics revealed significant influence of bioclimatic parameters on CT_min_ (*p* < 0.0001). It was directly related to the mean diurnal temperature range (BIO2), isothermality (BIO3), and minimum temperatures (BIO6) (Table 3, Figure 2, Figure S4). 

By contrast, for CT_max_, we found highly significant relationships with all three parameters: KCC, species, and body mass. Species had the highest *F*-quotient, but also KCC showed significant influence (Table 3). A generalized linear model (GLM) for CT_max_ showed highly significant correlations both for the entire model as well as for every single bioclimatic parameter. Temperature seasonality (BIO4) and minimum temperature of coldest month (BIO6) seemed to have a lesser but still significant effect. For details, see Table 3. Figure 2 shows the correlations of CT_min_ and CT_max_ with latitude and climatic parameters BIO1, and BIO4 to BIO7.

### 3.2. Correlation with Derived Physiological Thermal Tolerance Parameters (TTB, TC, TH) 

**Figure 3:**
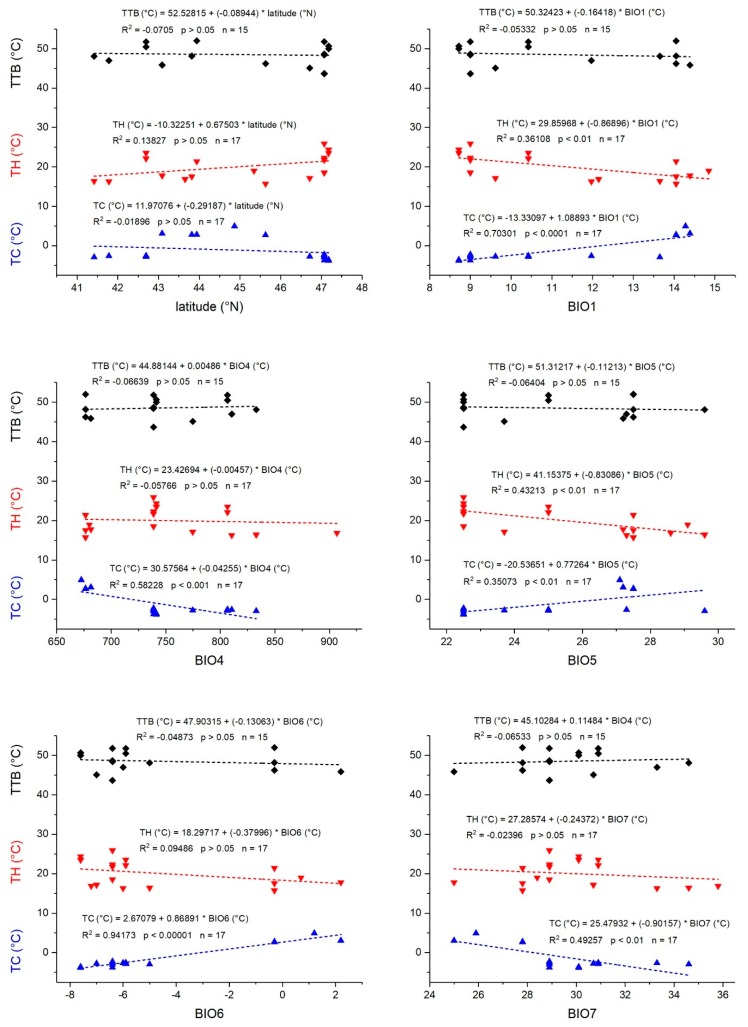
Physiological parameters thermal tolerance breadth (TTB, black), and heat and cold tolerance (TH, red; TC, blue) derived from CT_max_ and CT_min_ (see Methods section) in dependence on latitude and bioclimatic variables (BIO1, BIO4–BIO7). Dots represent means of individual species at certain sample sites. See also Table 2 and Table S2.

Thermal tolerance breadth (TTB) ranged from 43.7 °C in *Scoloposthetus pictus* (Graz, AT) to 52.0 °C in *Melanocoryphus albomaculatus* (Pistoia, IT) (Table 2). It was influenced by species (ANOVA, df = 7, *F* = 11.37, *p* = 0.0357) but did not correlate with KCC or mass. A GLM for all bioclimatic parameters showed a (weak) dependency on BIO1, BIO4, BIO5, and BIO6 after successive removal of non-significant parameters (Table 4, see Figure 3 for correlations of single parameters).

**Figure 4:**
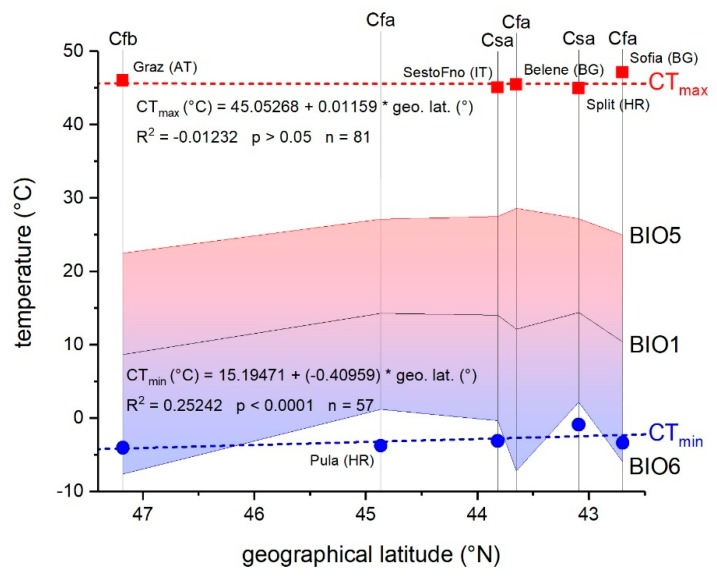
Thermal tolerance breadth (CT_max_ − CT_min_) of *Pyrrhocoris apterus* as well as bioclimatic variables BIO1 (mean annual temperature), BIO5 (maximum temperature of warmest month), and BIO6 (minimum temperature of coldest month) at the sample sites in dependence on geographical latitude.

Cold tolerance (TC = BIO6 − CT_min_) was highest in *Rhyparochromus vulgaris* (−3.8 °C, Gschwendt, AT) and lowest in *Pyrrhocoris apterus* (5.0 °C, Pula, HR) (Table 2). In Austria and Bulgaria, BIO6 (minimum temperature of the coldest month) was lower than the tested species’ CT_min_ (see Figure 4 for *P. apterus*). TC did not correlate with KCC, species, or mass, but with some bioclimatic parameters (GLM, df = 4, *F* = 224.56, *p* < 0.0001). BIO1 to BIO3, and BIO6 fitted the model significantly after removal of non-significant parameters (Table 4).

Heat tolerance (TH = CT_max_ − BIO5) was lowest in *Orsillus maculatus* (15.7 °C, Tregnago, IT) and highest in *Oxycarenus lavaterae* (25.9 °C, Graz, AT) (Table 2). It was not dependent on KCC, species, mass (ANOVA, *p* > 0.05 for all), or bioclimatic factors (GLM, df = 7, *p* > 0.05) (Table 4). In all species from all locations, CT_max_ was considerably higher than the maximal temperature of the warmest month (BIO5) (compare Figure 4). 

### 3.3. Testing for Phylogenetic Signal

A strong phylogenetic signal was detected in body mass (Table 5), which generally can be explained by small mass differences between several species (compare Figure S2). We found no significant phylogenetic signal in the tested physiological parameters using Pagel’s λ for critical thermal minimum (CT_min_), temperature breath (TTB), heat tolerance (TH), and cold tolerance (TC) (λ = 0 with distinct values for hypothesis and null hypothesis). For the critical thermal maximum (CT_max_), a phylogenetic signal was indicated (Pagel’s λ = 1, *p*_(λ=1)_ = 1) but the null hypothesis of no signal showed also a certain probability (*p*_(λ=0)_ = 0.235).

## 4. Discussion

True bugs are an abundant and successful insect group, capable of populating a wide variety of habitats. The species covered in this paper are closely related, of the families Lygaeidae and Pyrrhocoridae, and colonized four Köppen–Geiger climate regions over a latitudinal range of almost 6°. 

Upper thermal limits in the tested bug species varied stronger (SD = 2.5 °C, range = 39.4–49.5 °C; total mean CT_max_ = 45.3 °C) than lower limits (SD = 1.4 °C, range = −4.6–2.1 °C; mean CT_min_ = −3.3 °C) (*p* < 0.001, *F*-test; see Table 2). Regarding the upper limits, this concurs with the findings of Kellermann et al. ([57], on fruit flies). However, the ability to withstand high temperatures (CT_max_) declines with an improved tolerance to low temperatures (CT_min_) in dung beetles [19]. In our seed bugs, CT_max_ correlated significantly with all studied climatic parameters (Table 3), except BIO7 (a derived bioclimatic parameter: BIO7 = BIO5 − BIO6), despite the moderate variation in latitude (6°) and thus climate variability. This finding demonstrates that climate has a strong impact on seed bug heat tolerance. Only temperature seasonality (BIO4) and minimum temperature of the coldest month (BIO6) had a less pronounced (though significant) effect on CT_max_. Besides bioclimatic parameters, mass showed a strong positive effect on CT_max_ (Table 3, Figure S3). This coincides with findings in beetles [58,59]. In other insects, however, results are not consistent. In ants, a positive correlation was reported [60,61] but also a decrease with mass [62,63]. In termites, a positive [64] or no correlation was reported [65]. 

Concerning the ability to tolerate cold, by contrast, the results are more diverse (Table 3). Although CT_min_ is influenced by bioclimatic variables in general, only some bioclimatic variables seemed to have a direct effect, that is, the minimum temperature of the coldest month (BIO6) and associated parameters (mean diurnal range, BIO2; isothermality, BIO3). This seems plausible, because minimum environmental temperatures strongly determine survival during cold seasons, when behavioral avoidance is restricted, and thus the evolutionary drive to withstand them by physiological adaptation is high. If one accepts this interpretation, it seems intelligible that the annual mean temperature (BIO1) has only a weak effect on CT_min_. Mass had no effect on the CT_min_ of seed bugs (Table 3, Figure S3). By contrast, in ants, a decrease with mass was reported [63]. In a seasonal comparison of two termite species, no effect of mass on CT_min_ was reported [65]. Raschmanová et al. [66] observed a significant decrease of cold resistance with increasing body length in Collembola species inhabiting soil and subterranean habitats.

Responses to temperatures below the favorable temperature range (i.e., cold stress) often rely on different physiological mechanisms than those to heat stress. They are decoupled evolutionarily, and therefore may change differently in the course of the species’ colonization of new areas [67]. Our findings support the hypothesis of decoupled thermal limits. Although cold tolerance (TC) correlated significantly with several bioclimatic parameters in a GLM analysis, heat tolerance (TH) did not (Table 4). This is in good accordance with a study of Addo-Bediako et al. [20], who found that upper thermal limits show little geographical variation, but the lower bounds of supercooling points and lower lethal temperatures do decline with latitude (and thus minimum temperature). In individual regression analyses (where other bioclimatic parameters were not included as covariates), cold tolerance (TC) depended on all tested bioclimatic parameters (Figure 3). On the one hand, it is plausible that with decreasing annual mean temperature (BIO1) and the minimum temperature of the coldest month (BIO6), the bugs adapted to endure lower temperatures (have a lower CT_min_; Figure 2). On the other hand, the observed negative values of cold tolerance (TC) under cold conditions (minimum temperature of the coldest month, BIO6; Figure 3) would indicate that the bugs were below their safe thermal conditions. This suggests that they seek out more favorable microclimatic environments in good time, that is, at higher ambient temperatures where unhindered mobility is guaranteed. The temperature in these microclimatic environments may well be higher than indicated by the macroclimatic variables [68]. By contrast, heat tolerance TH, which represents the difference of CT_max_ to the maximum temperature of the warmest month (BIO5), depended only on the annual mean temperature (BIO1) and the maximum temperature of the warmest month (BIO5) (Figure 3, Table S2). A decreasing heat tolerance with these bioclimatic variables (BIO1, BIO5) indicates a decrease of the upper thermal safety margin (compare Figure 4). It remains unclear, however, whether this occurs because of physiological restraints (i.e., inability for a higher CT_max_) or a lack of need to increase CT_max_ further (because the thermal safety range is already sufficient). 

Both cold and heat tolerance (TC, TH) did not correlate with latitude (Figure 3). This could have been due to the narrow latitudinal range of 6°, as already mentioned above. Our results demonstrated that the investigation of variables directly affecting the studied organisms, instead of surrogate variables such as latitude or elevation, should increase the ability to understand the mechanisms driving animal distribution and biodiversity (see also [69]).

It has to be kept in mind, however, that our analysis does not include all ecological factors relevant for survival of unfavorable conditions. Mean minimum temperatures of the coldest month (BIO6) in Austria and Bulgaria were lower than the species’ CT_min_, resulting in negative values of cold tolerance (TC) (Figure 4). Here, the overwintering individuals have to seek out microhabitats that provide a more benign microclimate, wherein they can endure cold periods [68,70,71]. However, if endured winter temperatures sink under the lethal temperatures of the bug species, colonization of such regions is not possible. This might explain the absence of, for instance, the Mediterranean *Orsillus maculatus* in colder regions of Europe, whereas *Orsillus depressus* thrives in these regions [43]. 

Heat tolerance (TH) in all species exceeded mean maximum temperatures of the warmest month (BIO5) in all locations (Table 2; by up to 25.9 °C in *Oxycarenus lavaterae* in Graz/AT), which coincides with the findings of Sunday et al. [24] in several ectotherms. As all individuals were sampled during the cold season (winter), an immediate acclimation to high temperatures is unlikely. However, as bioclimatic parameters were the climatological normal from 1970 to 2000, absolute maximum temperatures were likely to be higher at some times, but not to the extent that they make permanent populations impossible.

The climate variability hypothesis states that a positive relationship exists between the breadth of thermal tolerance (TTB, the degree of eurythermy) and the level of climatic variability experienced by taxa with increasing latitude, especially in terrestrial ectotherms [16,72]. However, on the basis of our results, we could not make a clear decision because there was no correlation with BIO7 (BIO5 − BIO6), the bioclimatic variable we assumed to characterize best the climatic variability, but there was a weak correlation with BIO4 (temperature seasonality) (Table 4). One reason for the ambiguity could be the small latitudinal range covered in this study and the resulting small climatic variability.

The presence of a phylogenetic signal, that is, when related species share similar thermal responses, may indicate constraints of their thermal niches, which dictate the environments in which they can persist [73], or it may indicate similar selection pressures and similar environmental effects [73,74,75]. In our assessed seed bug species, kinship seems to have played a lesser, or at least no unambiguous, role in the adaptation to high environmental temperatures, and no role in low temperature adaptation (Table 5). This concurs with the findings of Teets and Hahn [76] on CT_min_ of *Drosophila*. Concerning CT_max_, it concurs with findings of Ayrinhac et al. [77] and Hoffmann et al. [78] regarding the upper thermal limits in *Drosophila*, of Terblanche et al. [79] in *Glossina pallidipes*, of Vorhees et al. [39] in *Culex tarsalis,* and of Hamblin et al. [80] in a variety of bee species. In other words, our results suggest that despite phylogenetic relatedness, the seed bugs differ in their CT_max_ because of adaptation to differing thermal environments, indicated by the comprehensive significant effects of bioclimatic variables on CT_max_ (Figure 2, Table 3 and Table 4).

In the face of global warming and the current “Mediterranaziation” of the Heteroptera fauna [81,82] in regions of previously unsuitable climatological (i.e., “too cold”) conditions, we suggest that seed bugs will not experience troubles to disperse towards the north. Temperature-induced longer (reproductive) seasons and shortened diapause may result in an increase of generations per year or/and increased overwintering success [83,84,85]. Although winter mortality may vary depending on parameters such as gender, microhabitat choice, as well as size and coloration [86,87,88], temperature seems to be the main factor. That which might hinder the true bugs’ dispersion could be the minimum environmental temperatures, which still can reach quite low values in Europe despite higher annual temperatures. Yukawa et al. [89] suggested the northward range expansion of *Nezara viridula* in Japan to be due to global warming, because the monthly mean temperature for January in the newly invaded areas exceeded the limit temperature of 5 °C, below which winter mortality increases by 15%–16.5% per 1 °C decrease in mean winter temperature [87,90,91]. Concerning the ability to invade colder areas in reaction to global temperature increase, we suggest a similar dispersion capacity of European seed bugs than predicted for *Nezara viridula* in Kyushu in Southern Japan [89].

## 5. Conclusions

Physiological traits such as lower and upper thermal limits play an important role in seed bug survival, reproduction, dispersion, and colonization under given environmental conditions. We suggest that their wide thermal breath and adaptive capacity have promoted their prosperity and dispersion. In this respect, there seems to be ample potential in the bugs for thriving and further proliferation, as well as invasion to new, hitherto unexploited areas of settlement, especially in the face of global warming.

## Figures and Tables

**Table 1:** Bug species sampled and their systematic order [6,43].

Species	Subfamily	Family	Suborder	Order
*Arocatus longiceps*	Lygaeinae	Lygaeidae	Heteroptera	Hemiptera
*Melanocoryphus albomaculatus*				
*Orsillus depressus*	Orsillinae			
*Orsillus maculatus*				
*Oxycarenus lavaterae*	Oxycareninae			
*Rhyparochromus vulgaris*	Rhyparochrominae			
*Scolopostethus pictus*				
*Pyrrhocoris apterus*		Pyrrhocoridae		

**Table 2:** Species, sample location and Köppen–Geiger climate classification (KCC), mean body mass with SD, critical thermal minima and maxima (CT_min_, CT_max_) with SD, thermal tolerance breadth (TTB), and heat and cold tolerance (TH, TC) of the true bugs assessed.

Species	Location - KCC	Lat (°N)/Long (°E)	Mass	CT_min_ ± SD		Mass	CT_max_ ± SD		TTB	TH	TC
			(mg)	(°C)	*n*	(mg)	(°C)	*n*	(°C)	(°C)	(°C)
*Arocatus longiceps*	Graz (AT) - Cfb	47.071/15.44	6.6 ± 1.1	−4.2 ± 0.4	8	6.7 ± 1.3	44.2 ± 0.5	30	48.4	21.7	−2.2
	Sofia (BG) - Cfa	42.696/23.334	6.8 ± 0.9	−3.1 ± 0.8	6	-	-	-	-	-	−2.8
	Kresna gorge (BG) - Cfa	41.783/23.155	3.6 ± 0.6	−3.4 ± 1.6	2	3.6 ± 1	43.6 ± 0.6	25	47.0	16.3	−2.6
*Melanocoryphus albomaculatus*	Pistoia (IT) - Cfa	43.939/10.849	14.4 ± 3.4	−3.1 ± 0.3	9	14.6 ± 2.6	48.9 ± 0.5	14	52.0	21.4	2.8
*Orsillus depressus*	Graz (AT) - Cfb	47.071/15.44	9.7 ± 1.8	−3.8 ± 0.4	9	9.2 ± 1.6	44.8 ± 0.8	10	48.6	22.3	−2.6
*Orsillus maculatus*	Tregnago (IT) - Cfb	45.629/11.095	15.2 ± 4.6	−3.0 ± 0.7	5	10.7 ± 2.4	43.2 ± 1.2	25	46.2	15.7	2.7
	Marino Pole (BG) - Bsk	41.419/23.331	11.5 ± 2.5	−2.1 ± 2.6	7	11.4 ± 2.7	46.0 ± 0.4	10	48.1	16.4	−2.9
*Oxycarenus lavaterae*	Sofia (BG) - Cfa	42.696/23.334	2.5 ± 0.8	−3.2 ± 2.0	8	3.1 ± 0.5	48.5 ± 0.3	19	51.7	23.5	−2.7
	Graz (AT) - Cfb	47.071/15.44	4.0 ± 0.7	−3.4 ± 2.1	8	3.4 ± 0.8	48.4 ± 0.2	19	51.8	25.9	−3.0
	Legnaro (IT) - Cfa	45.346/11.964	-	-	-	4.7 ± 0.7	48.1 ± 0.4	32	-	19.0	-
*Rhyparochromus vulgaris*	Gschwendt (AT) - Cfb	47.179/15.573	15.1 ± 2.5	−3.8 ± 0.5	10	17.1 ± 3.5	46.9 ± 0.4	10	50.7	24.4	−3.8
*Scolopostethus pictus*	Radkersburg (AT) - Cfb	46.714/15.998	2.8 ± 0.7	−4.3 ± 0.2	9	3.5 ± 0.6	40.8 ± 0.6	29	45.1	17.1	−2.7
	Graz (AT) – Cfb	47.071/15.44	3.9 ± 0.8	−2.7 ± 0.4	9	3.3 ± 0.6	41 ± 0.4.0	9	43.7	18.5	−3.7
*Pyrrhocoris apterus*	Graz (AT) - Cfb	47.179/15.573	55.9 ± 9.3	−4.0 ± 0.6	21	53.2 ± 13.4	46.0 ± 0.5	10	50.0	23.5	−3.6
	SestoFno/FI (IT) - Csa	43.818/11.204	24.5 ± 2.8	−3.1 ± 1.4	8	39.0 ± 8.5	45.0 ± 0.5	19	48.1	17.5	2.8
	Belene (BG) - Cfa	43.652/25.129	-	-	-	25.4 ± 5	45.5 ± 0.6	32	-	16.9	-
	Sofia (BG) - Cfa	42.696/23.334	29.5 ± 6.7	−3.4 ± 1.6	9	47.3 ± 8.4	47.1 ± 0.2	10	50.4	22.1	−2.5
	Split (HR) - Csa	43.09/16.752	39.0 ± 6.4	−0.9 ± 0.6	10	29.8 ± 2.9	45.0 ± 0.4	10	45.9	17.8	3.1
	Pula (HR) - Cfa	44.867/13.85	31.6 ± 3.4	−3.8 ± 0.7	9	-	-	-	-	-	5.0

**Table 3:** Statistical analysis of CT_min_ and CT_max_ on Köppen–Geiger climate classification (KCC), species, body mass, (via multifactorial ANOVA), and bioclimatic parameters (via general linear model (GLM)).

	CT_min_			CT_max_		
	df	*F*-Quotient	*p*-Value	df	*F*-Quotient	*p*-Value
KCC	3	10.46	<0.0001	4	46.7	<0.0001
Species	7	0.8	0.5903	7	508.36	<0.0001
Mass	1	1.08	0.3002	1	26.43	<0.0001
GLM for all bioclimatic parameters	7	10.37	<0.0001	7	36.01	<0.0001
BIO1 = annual mean temperature	1	6.43	0.0123	1	48.27	<0.0001
BIO2 = mean diurnal range	1	15.23	<0.001	1	40.57	<0.0001
BIO3 = isothermality ((BIO2/BIO7) × 100)	1	17.60	<0.0001	1	48.69	<0.0001
BIO4 = temperature seasonality (SD × 100)	1	0.76	0.3863	1	9.32	<0.005
BIO5 = max temperature of warmest month	1	1.14	0.2875	1	50.04	<0.0001
BIO6 = min temperature of coldest month	1	20.43	<0.0001	1	16.32	<0.001
BIO12 = annual precipitation	1	2.50	0.1163	1	46.84	<0.0001

**Table 4:** Statistical analysis of TTB (thermal tolerance breadth), TC (cold tolerance), and TH (heat tolerance) on Köppen–Geiger climate classification (KCC), species, body mass, (via multifactorial ANOVA), and bioclimatic parameters (via GLM). Empty fields indicate non-significant parameters excluded from the model. The effect of temperature annual range (BIO7) on thermal tolerance breadth (TTB) had to be calculated independently because of inadmissible interactions (BIO7 = BIO5 − BIO6).

	TTB			TC			TH		
	df	*F*-Quotient	*p*-Value	df	*F*-Quotient	*p*-Value	df	*F*-Quotient	*p*-Value
KCC	3	0.86	0.6225	3	1.22	0.3939	3	2.07	0.2223
Species	7	11.37	0.0357	7	1.09	0.4788	7	3.87	0.0776
Mass	1	2.24	0.2316	1	0.7	0.4398	1	1.36	0.2959
GLM	4	1.47	0.2836	4	224.56	<0.0001	7	1.65	0.2379
BIO1	1	5.42	0.0421	1	23.97	<0.0005	-	-	-
BIO2	-	-	-	1	26.00	<0.0005	-	-	-
BIO3	-	-	-	1	28.40	<0.0005	-	-	-
BIO4	1	5.21	0.0456	-	-	-	-	-	-
BIO5	1	5.44	0.0419	-	-	-	-	-	-
BIO6	1	5.00	0.0494	1	18.93	<0.001	-	-	-
BIO12	-	-	-	-	-	-	-	-	-
BIO7	1	0.14	0.7135	-	-	-	-	-	-

**Table 5:** Statistical analysis of phylogenetic signal in fresh body mass, CT_min_, CT_max_, TTB (thermal tolerance breadth), TC (cold tolerance), and TH (heat tolerance). Estimation of phylogenetic signals for single traits with Pagel’s λ: *p_(_*_λ=0)_ states the significance level of “no phylogenetic signal”, and P_(λ=1)_ the significance level of “a strong phylogenetic signal. In the case of body mass, for example, *p*_(λ=0)_ < 0.001 means that the hypothesis of no phylogenetic signal was discarded, and P_(λ=1)_ = 1 means that the hypothesis of a strong phylogenetic signal was confirmed (i.e., is not discarded).

	Pagel’s λ	*p*_(λ=0)_	*p*_(λ=1)_
Body mass (mg)	1	<0.001	1
CT_max_ (°C)	1	0.235	1
CT_min_ (°C)	0	1	0.008
TTB (°C)	0	1	0.197
TH (°C)	0	1	0.011
TC (°C)	0	1	0.069

## Supplementary Materials

The following are available online at https://www.mdpi.com/2075-4450/11/3/197/s1, Figure S1: Theoretical cladogram. Figure S2: Correlation of CT_min_ and CT_max_ with fresh body mass. Figure S3: Correlation of physiological parameters with bioclimatic variables. Table S1: Species, sample date, sample location, KCC, and bioclimatic parameters of the true bugs assessed. Table S2: Statistical results.

## Abbreviations

*KCC Köppen–Geiger climate classification (climate, precipitation, temperature)*	
Bsk	arid, summer dry, cold arid
Cfa	warm temperate, fully humid, hot summer
Cfb	warm temperate, fully humid, warm summer
Csa	warm temperate, summer dry, hot summer
*Bioclimatic variables (mean values 1970–2000)*	
BIO1	annual mean temperature
BIO2	mean diurnal range (mean of monthly (max temp − min temp))
BIO3	isothermality (BIO2/BIO7 × 100)
BIO4	temperature seasonality (standard deviation × 100)
BIO5	max temperature of warmest month
BIO6	min temperature of coldest month
BIO7	temperature annual range (BIO5 − BIO6)
BIO12	annual precipitation
*Physiological parameters*	
CT_min_	critical thermal minimum
CT_max_	critical thermal maximum
TTB	thermal tolerance breadth (CT_max_ − CT_min_)
TC	cold tolerance (BIO6 − CT_min_)
TH	heat tolerance (CT_max_ − BIO5)
