# Supplementary material for: Temperature Tolerance and Thermal Environment of European Seed Bugs
Source: Insects. 2020 Mar 20;11(3):197. doi: 10.3390/insects11030197 (PMC7143385; doi:10.3390/insects11030197)
Supplement: Supplementary file 1 [file insects-11-00197-s001.pdf]

insects

Article

Temperature tolerance and thermal environment of European seed bugs

Helmut Käfer [1,*], Helmut Kovac [1,*], Nikolay Simov [2], Andrea Battisti [3], Bettina Erregger [1,4], Arne K. D. Schmidt [1,5] and Anton Stabentheiner [1,*]

1 Institute of Biology, University of Graz, Austria
2 National Museum of Natural History, Sofia, Bulgaria
3 School of Agricultural Sciences and Veterinary Medicine, University of Padova, Italy
4 Institute of Animal Nutrition, Livestock Products, and Nutrition Physiology, University of Natural Resources and Life Sciences, Vienna, Austria
5 AGES; The Austrian Agency for Health and Food Safety, Vienna, Austria
* Correspondence: helmut.kaefer@uni-graz.at, helmut.kovac@uni-graz.at, anton.stabentheiner@uni-graz.at

Received: date; Accepted: date; Published: date

Supplementary material:

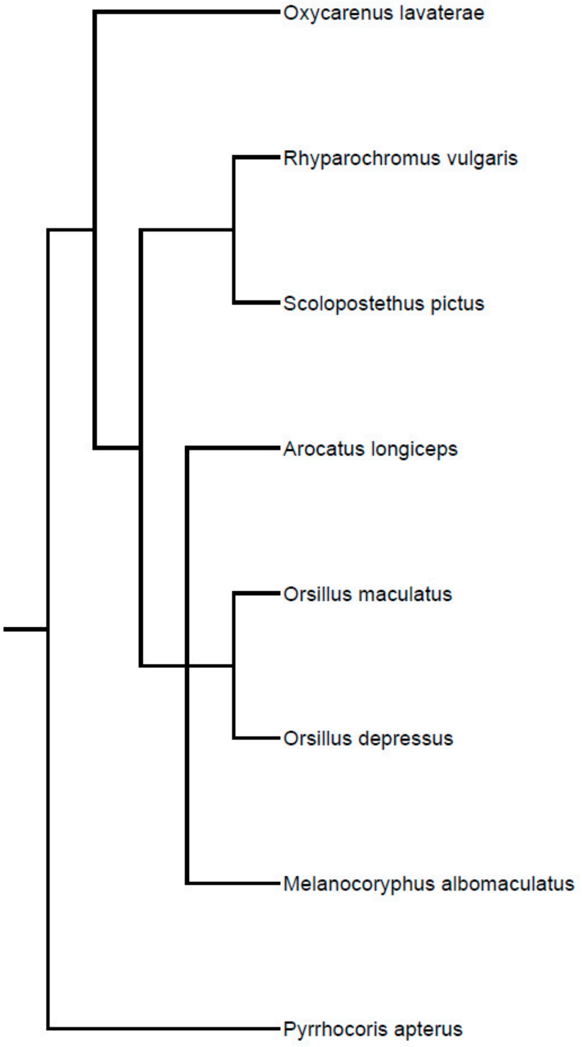

Figure S1. Theoretical cladogram for our test for a phylogenetic signal. Taxonomy based on [6, 42].

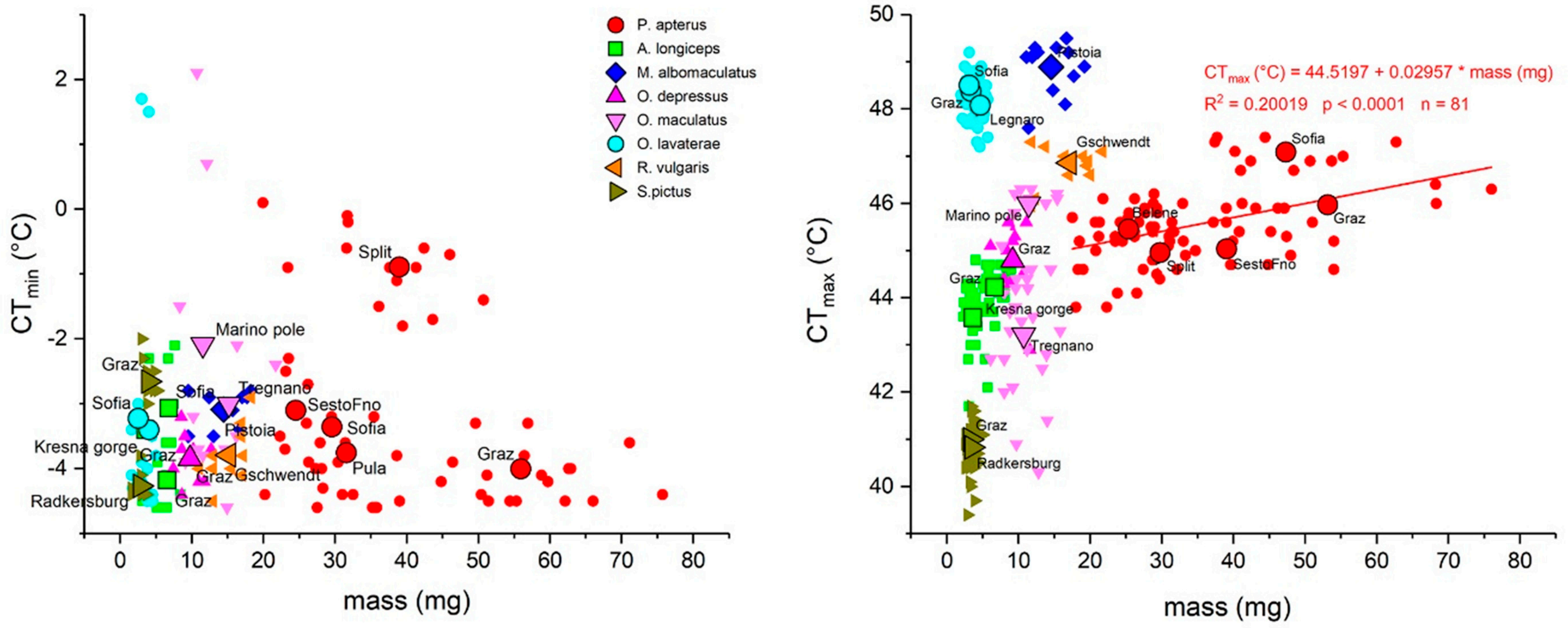

Figure S2. Correlation of CT_{min} and CT_{max} with fresh body mass in single bug species. Small symbols are data points of individuals, large, black-framed symbols represent bug species at sample points. Species are color coded. For *Pyrrhocoris apterus* the correlation of CT_{max} on body mass is shown (regression line).

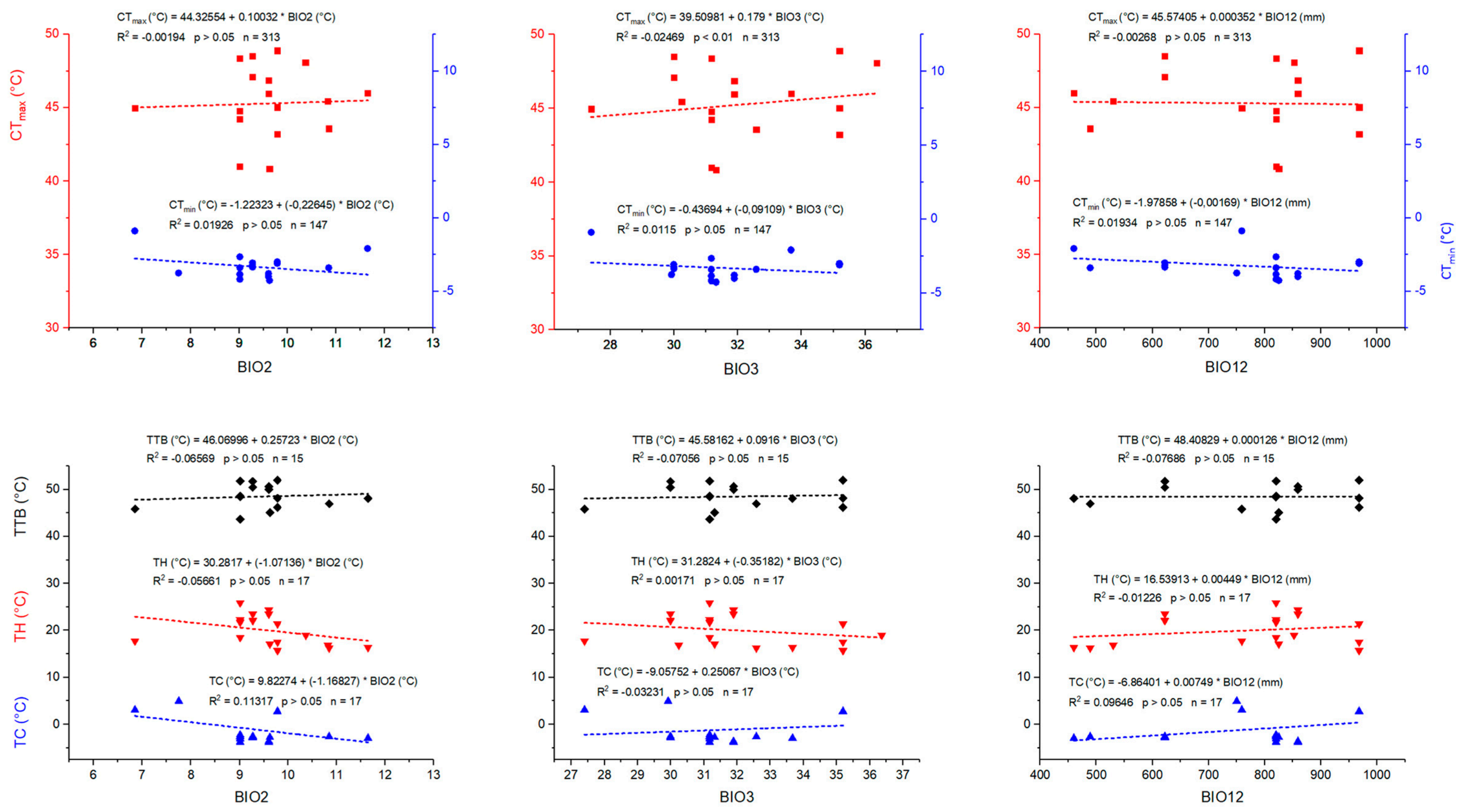

Figure S3. Correlation of physiological parameters CT_{min}, CT_{max}, TTB (thermal tolerance breadth), TC (cold tolerance), and TH (heat tolerance) with latitude and bioclimatic variables (BIO2, BIO3, BIO12; from [45]; WorldClim 2; variables are the average from 1970-2000). Dots represent means of individual species at certain sample sites. See also Table 2 and Table S2.

Table S1. Species, sample date, sample location, altitude (ASL), Köppen-Geiger climate classification group (KCC), and bioclimatic parameters of the true bugs assessed.

**Species**	**Sample date**		**Location**	**Latitude**	**Longitude**	**ASL**	**KCC**	**BIO1**	**BIO2**	**BIO3**	**BIO4**	**BIO5**	**BIO6**	**BIO7**	**BIO12**
	**CT_{max}**	**CT_{min}**		(°N)	(°E)	**(m)**		**(°C)**	**(°C)**	**(°C)**	**(°C)**	**(°C)**	**(°C)**	**(°C)**	(mm)
Arocatus longiceps	08.11.2013	21.11.2014	Graz (AT)	47.071	15.44	367	Cfb	9.0	9.0	31.2	738.8	22.5	-6.4	28.9	820
	-	17.11.2014	Sofia (BG)	42.696	23.334	560	Cfa	10.4	9.3	30.0	806.6	25.0	-5.9	30.9	622
	06.01.2014	05.12.2014	Kresna gorge (BG)	41.783	23.155	224	Cfa	12.0	10.9	32.6	810.5	27.3	-6.0	33.3	489
Melanocoryphus albomaculatus	04.11.2013	26.10.2014	Pistoia (IT)	43.939	10.849	221	Cfa	14.1	9.8	35.2	676.5	27.5	-0.3	27.8	968
Orsillus depressus	09.10.2018	30.09.2014	Graz (AT)	47.071	15.44	367	Cfb	9.0	9.0	31.2	738.8	22.5	-6.4	28.9	820
Orsillus maculatus	19.01.2014	16.01.2016	Tregnago (IT)	45.629	11.095	1230	Cfb	14.1	9.8	35.2	676.5	27.5	-0.3	27.8	968
	17.10.2018	29.11.2014	Marino pole (BG)	41.419	23.331	135	Bsk	13.7	11.7	33.7	832.9	29.6	-5.0	34.6	460
Oxycarenus lavaterae	18.11.2013	28.10.2014	Sofia (BG)	42.696	23.334	560	Cfa	10.4	9.3	30.0	806.6	25.0	-5.9	30.9	622
	16.12.2013	06.11.2014	Graz (AT)	47.071	15.44	367	Cfb	9.0	9.0	31.2	738.8	22.5	-6.4	28.9	820
	20.01.2014	-	Legnaro (IT)	45.346	11.964	8	Cfa	14.9	10.4	36.4	679.9	29.1	0.7	28.4	852
Rhyparochromus vulgaris	31.10.2013	19.09.2014	Gschwendt (AT)	47.179	15.573	523	Cfb	8.7	9.6	31.9	741.4	22.5	-7.6	30.1	859
Scolopostethus pictus	06.10.2013	22.11.2014	Radkersburg (AT)	46.714	15.998	208	Cfb	9.6	9.6	31.3	774.7	23.7	-7.0	30.7	825
	06.10.2013	01.10.2014	Graz (AT)	47.071	15.44	367	Cfb	9.0	9.0	31.2	738.8	22.5	-6.4	28.9	820
Pyrrhocoris apterus	07.11.2013	19.09.2014	Graz (AT)	47.179	15.573	523	Cfb	8.7	9.6	31.9	741.4	22.5	-7.6	30.1	859
	24.11.2013	04.11.2014	SestoFno (IT)	43.818	11.204	49	Csa	14.1	9.8	35.2	676.5	27.5	-0.3	27.8	968
	24.11.2013	-	Belene (BG)	43.652	25.129	28	Cfa	12.2	10.8	30.2	906.9	28.6	-7.2	35.8	530
	08.10.2018	28.10.2014	Sofia (BG)	42.696	23.334	560	Cfa	10.4	9.3	30.0	806.6	25.0	-5.9	30.9	622
	25.10.2016	14.09.2018	Split (HR)	43.09	16.752	98	Csa	14.4	6.9	27.4	681.4	27.2	2.2	25.0	759
	-	24.10.2014	Pula (HR)	44.867	13.85	15	Cfa	14.3	7.8	29.9	672.6	27.1	1.2	25.9	750

Table S2. Dependencies of physiological parameters, CT_{min}, CT_{max}, TTB (thermal tolerance breadth), TC (cold tolerance), and TH (heat tolerance), on latitude and bioclimatological parameters - Statistical results (ANOVA). Significant dependencies are shown in red. Compare also Figure 2, Figure 3, Figure S3.

p	CT_{max}	CT_{min}	TTB	TH	TC
Latitude	< 0.001	< 0.0001	> 0.05	> 0.05	> 0.05
BIO1	< 0.0001	< 0.00001	> 0.05	< 0.01	< 0.0001
BIO2	> 0.05	> 0.05	> 0.05	> 0.05	> 0.05
BIO3	< 0.01	> 0.05	> 0.05	> 0.05	> 0.05
BIO4	> 0.05	> 0.05	> 0.05	> 0.05	< 0.001
BIO5	< 0.0001	< 0.00001	> 0.05	< 0.01	< 0.01
BIO6	< 0.00001	< 0.00001	> 0.05	> 0.05	< 0.00001
BIO7	> 0.05	> 0.05	> 0.05	> 0.05	< 0.01
BIO12	> 0.05	> 0.05	> 0.05	> 0.05	> 0.05

R^2	CT_{max}	CT_{min}	TTB	TH	TC
Latitude	0.0439	0.1405	-0.0705	0.1383	-0.019
BIO1	0.0512	0.1395	-0.0533	0.3611	0.703
BIO2	-0.0019	0.0193	-0.0657	-0.0566	0.1132
BIO3	-0.0247	0.0115	-0.0706	0.0017	-0.0323
BIO4	0.0067	0.0013	-0.0664	-0.0577	0.5823
BIO5	0.0461	0.1362	-0.064	0.4321	0.3507
BIO6	0.0605	0.1275	-0.0487	0.0949	0.9417
BIO7	0.005	0.0151	-0.0653	-0.024	0.4926
BIO12	-0.0027	0.0193	-0.0769	-0.0123	0.0965

© 2020 by the authors. Submitted for possible open access publication under the terms and conditions of the Creative Commons Attribution (CC BY) license (http://creativecommons.org/licenses/by/4.0/).
